# Senescence-like phenotype in post-mitotic cells of mice entering middle age

**DOI:** 10.18632/aging.103637

**Published:** 2020-07-31

**Authors:** Marco Raffaele, Kristina Kovacovicova, Francesca Bonomini, Rita Rezzani, Jan Frohlich, Manlio Vinciguerra

**Affiliations:** 1 International Clinical Research Center (FNUSA-ICRC), St’ Anne University Hospital, Brno, Czech Republic; 2 Anatomy and Physiopathology Division, Department of Clinical and Experimental Sciences, University of Brescia, Brescia, Italy; 3 Interdepartmental University Center of Research “Adaption and Regeneration of Tissues and Organs-(ARTO)”, University of Brescia, Brescia, Italy; 4 Department of Histology and Embryology, Faculty of Medicine, Masaryk University, Brno, Czech Republic; 5 Equal contribution

**Keywords:** senescence, mice, markers

## Abstract

Staining mice tissues for β-galactosidase activity is a fundamental tool to detect age- or disease-associated cellular senescence. However, reported analyses of positivity for senescence-associated β-galactosidase activity or for other markers of senescence in post-mitotic cells of healthy murine tissues have been fragmentary or inconclusive. Here, we attempted to independently deepen this knowledge using multiple senescence markers within the same cells of wild type mice entering middle age (9 months of age). A histochemistry protocol for the pH-dependent detection of β-galactosidase activity in several tissues was used. At pH 6, routinely utilized to detect senescence-associated β-galactosidase activity, only specific cellular populations in the mouse body (including Purkinje cells and choroid plexus in the central nervous system) were detected as strongly positive for β-galactosidase activity. These post-mitotic cells were also positive for other established markers of senescence (p16, p21 and DPP4), detected by immunofluorescence, confirming a potential senescent phenotype. These data might contribute to understanding the functional relation between the senescence-associated β-galactosidase activity and senescence markers in post-mitotic cells in absence of disease or advanced aging.

## INTRODUCTION

Cellular senescence is a cell state implicated in various physiological processes and also in many age-related diseases [1], described for the first time by Hayflick in 1961 [2]. In 2019, the International Cell Senescence Association (ICSA) released a consensus paper on the definition of what constitutes senescent cells: cell senescence is regarded as a state triggered by stress or certain physiological processes, characterized by a stable cell-cycle arrest with secretory features, macromolecular damage, and altered energy metabolism [1]. Conversely, many criteria that define senescent cells have also been observed in a wide range of post-mitotic cells, suggesting that senescence as a stress response can occur in non-dividing cells temporally uncoupled from cell cycle arrest [3, 4]. Rapid gain of interest in cellular senescence is rising from the possibility of therapeutically targeting it to improve healthy aging and age-related disease, using among others drugs called senolytics [5, 6], and from the creation a systematic and comprehensive approach to the classification and staging of organismal senescence in order to guide aging science policy and gerontology practice [7].

There is currently no single marker with absolute specificity for senescent cells. Some markers have more universal validity while others are related to specific senescent cell types. ICSA advised multi-marker approach, which combines broad and more specific markers for robust detection of senescent cells in tissues. One of the most frequently used marker of cell senescence is the activity of senescence-associated beta-galactosidase (SA-β-gal), hydrolase enzyme that catalyzes the hydrolysis of β-galactosides into monosaccharides [8]. pH is a classic and fundamental factor to discriminate SA-β-gal – operating at pH 6, from bacterial β-Gal – operating at pH 7.4, and from the endogenous β-gal - operating at pH 3–5 [9, 10]. More specific markers include nuclear proteins (*i.e.*, p16, p21) and senescence-associated heterochromatin foci (SAHF), which are specialized domains of facultative heterochromatin that contribute to silencing of proliferation-promoting genes in senescent cells [1, 11, 12]. Moreover, senescent cells are characterized by a secretory associated secretory phenotype (SASP, which includes various interleukins, chemokines, growth factors, inflammatory molecules, ligands and insoluble factors), which can help to confirm the senescent phenotype [1]. SA-β-gal was the first marker allowing the identification of senescent cells in culture and mammalian tissues, and connecting senescence with aging [13, 14]. Since 1995, the wide use of SA-β-gal to study senescence in human or mice tissues *in situ* has been accompanied by controversies and technical challenges. It has been proposed that SA-β-Gal staining does not depend on age but on the presence of certain pathologies and on the proliferative status of the cells studied, appearing even in “young” cells as long as they are not proliferative [15–17].

In this respect, while senescent features have been found to be activated in a range of post-mitotic cells, independent multi-marker integration and confirmation of these results is still lacking for most of them [3, 4].

## RESULTS

**Figure 1:**
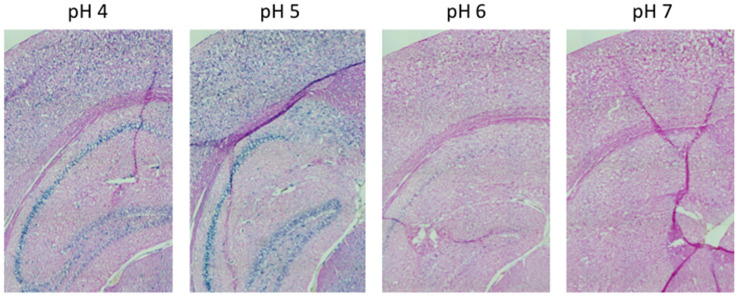
**pH-dependent (pH 4 to pH 7) β-gal activity in frozen sections of 9 months old C57/Bl6J mice hippocampus.** Nuclear Fast Red was used for counterstaining. At pH 6, specific for SA-β-gal, no marked β-gal activity is evident. Representative images from 3 different mice are shown.

**Figure 2:**
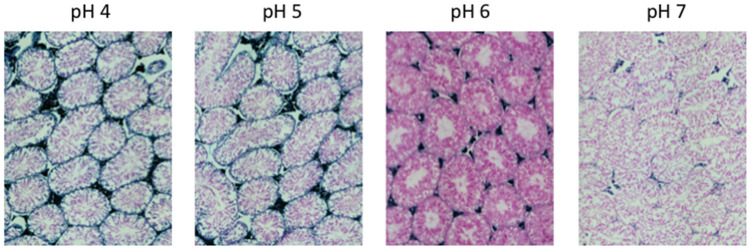
**pH-dependent (pH 4 to pH 7) β-gal activity in frozen sections of 9 months old C57/Bl6J mice testes.** Nuclear Fast Red was used for counterstaining. At pH 6, specific for SA-β-gal, no marked β-gal activity is evident. Representative images from 3 different mice are shown.

**Figure 3:**
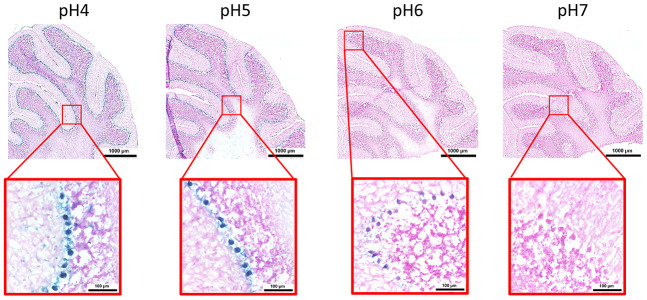
**pH-dependent (pH 4 to pH 7) β-gal activity in frozen sections of mouse cerebellum.** 9 months old C57/Bl6J. Nuclear Fast Red was used for counterstaining. At pH 6, specific for SA-β-gal, bluish color from β-gal activity is evident specifically in the Purkinje cell layer. Representative images from 3 different mice are shown.

**Figure 4:**
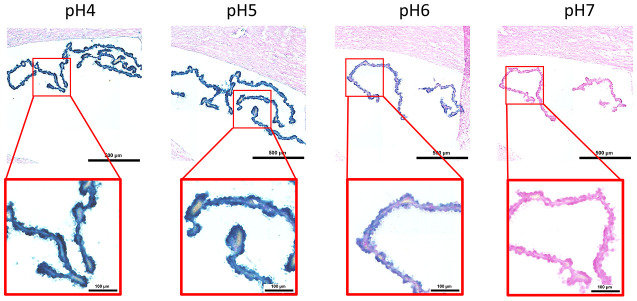
**pH-dependent (pH 4 to pH 7) β-gal activity in frozen sections of mouse choroid plexus.** 9 months old C57/Bl6J. Nuclear Fast Red was used for counterstaining. At pH 6, specific for SA-β-gal, bluish color from β-gal activity is evident specifically in ependymal cells in the choroid plexus. Representative images from 3 different mice are shown.

**Figure 5:**
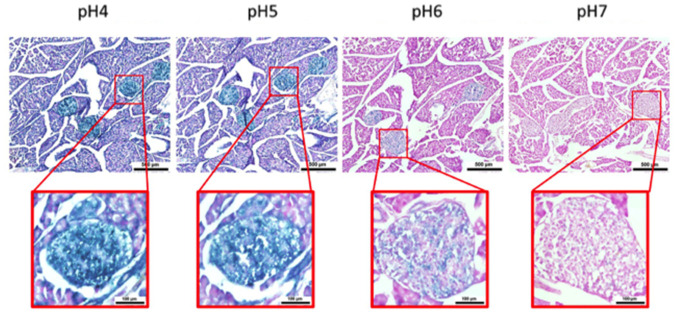
**pH-dependent (pH 4 to pH 7) β-gal activity in frozen sections of 9 months old C57/Bl6J mouse pancreas.** Nuclear Fast Red was used for counterstaining. At pH 6, specific for SA-β-gal, bluish color from β-gal activity is evident specifically in pancreatic islets. Representative images from 3 different mice are shown.

**Figure 6:**
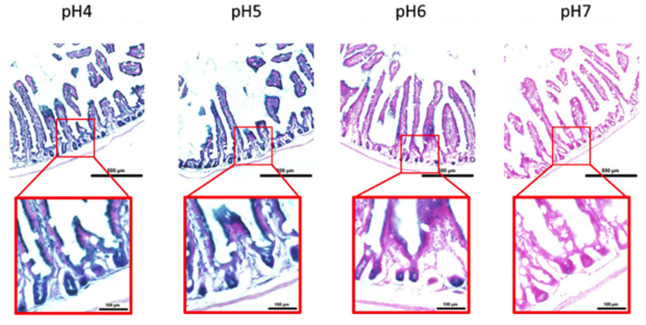
**pH-dependent (pH 4 to pH 7) β-gal activity in frozen sections of 9 months old C57/Bl6J mouse intestine.** Nuclear Fast Red was used for counterstaining. At pH 6, specific for SA- β-gal, bluish color from β-gal activity is evident specifically in cells located basally in the intestinal crypts. Representative images from 3 different mice are shown.

**Figure 7:**
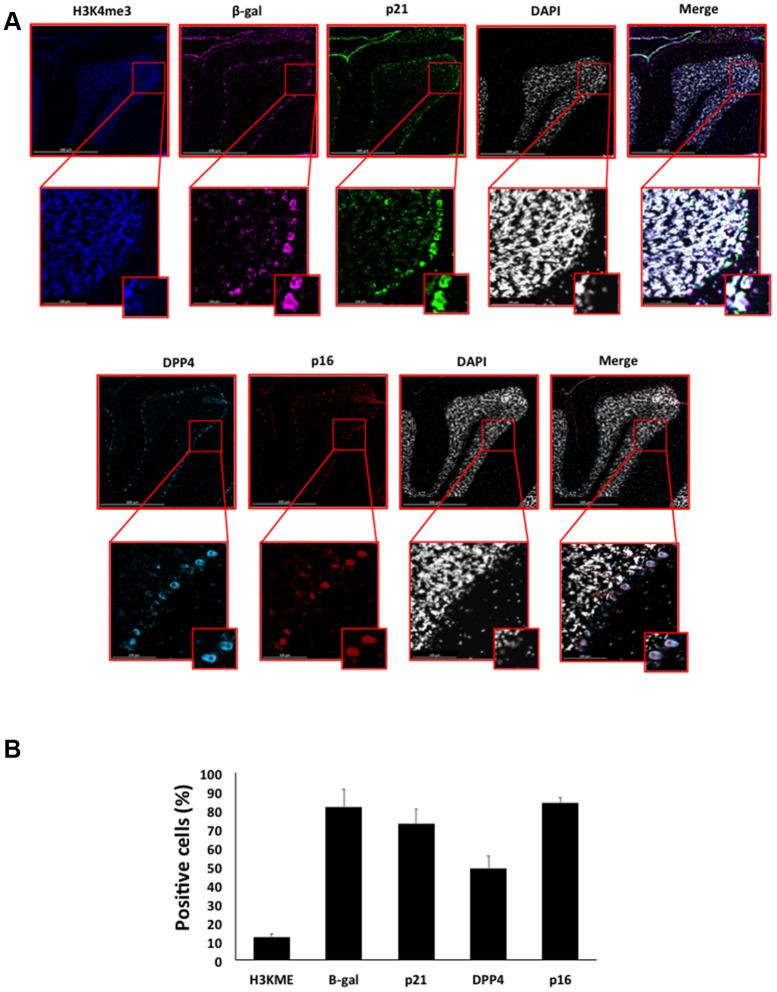
(**A**) Immunofluorescent staining of senescence markers in Purkinje cells of 9 months old C57/Bl6J mouse cerebellar cortex. Images display H3K4me3 (blue), β-gal (purple), p21 (green), DPP4 (cyan) p16 (red) and DAPI-stained nuclei (white) fluorescence signals. The corresponding multichannel overlaid images are shown in the right column. All these markers, except H3K4me3, show an increased localization and expression in Purkinje cells. Representative images from 3 different mice are shown. (**B**) Frequency of positive cells for each marker as in (**A**), indicated in %.

**Figure 8:**
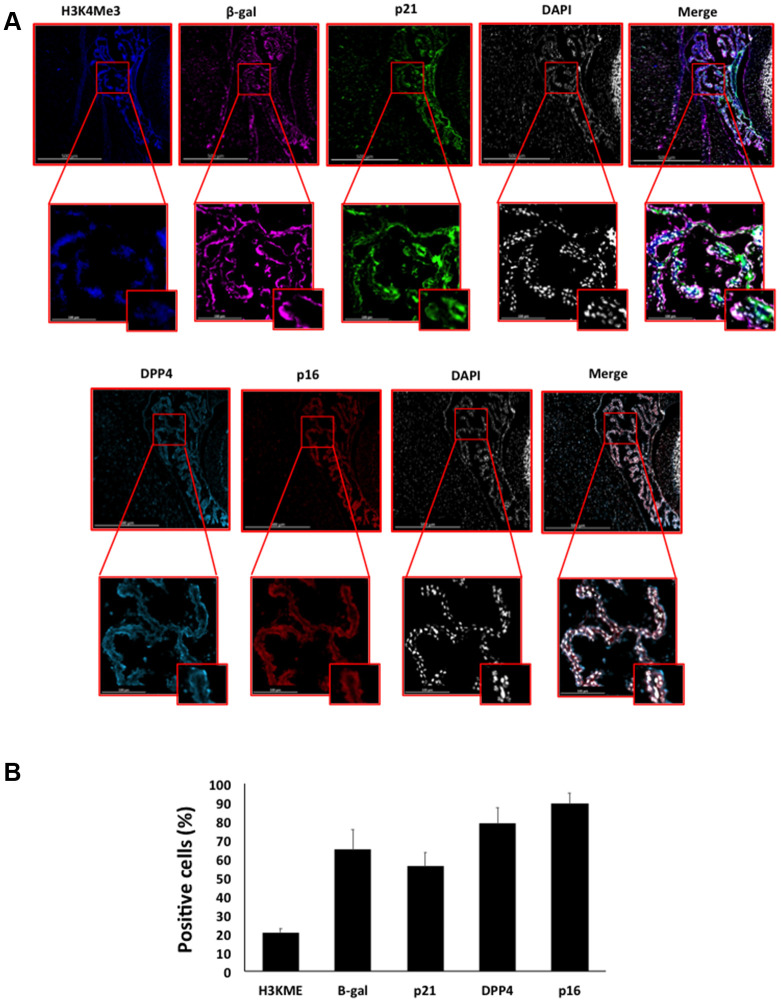
(**A**) Immunofluorescent staining of senescence markers of 9 months old C57/Bl6J mouse choroid plexus. Images display H3K4me3 (blue), β-gal (purple), p21 (green), DPP4 (cyan) p16 (red) and DAPI-stained nuclei (white) fluorescence signals. The corresponding multichannel overlaid images are shown in the right column. All these markers show an increased localization and expression in choroid plexus cells. Representative images from 3 different mice are shown. (**B**) Frequency of positive cells for each marker as in (**A**), indicated in %.

Here, we performed an analysis for SA-β-gal (pH 6.0) staining on a range of tissues of healthy mice entering middle age (9 months old): heart muscle, skeletal muscle, bone-femur, brain-cortex, brain-hippocampus, brain-cerebellum, choroid plexus, lymph nodes, intestine, pancreas, kidney, visceral adipose tissue, liver and lungs; according to a well-established protocol [18, 19]. The majority of tissues analyzed exhibited cellular populations with strong diffuse β-gal positivity at low pH values (4 to 5), which fainted or disappeared at pH 6 to 7, indicating a non SA-β-gal activity: examples of tissues following this pattern included the hippocampus and the testes (Figures 1 and 2). Conversely, we report strong SA-β-gal staining (pH 6) in cerebellum (Figure 3), choroid plexus (Figure 4), pancreatic islets (Figure 5) and basal/stem cells in the intestinal crypts (Figure 6) of adult wild type mice. A distinct β-galactosidase staining was detected from pH 4 to SA-β-gal-associated pH 6 specifically for the Purkinje cell layer, containing the large GABAergic neurons constituting the output of all motor coordination in the cerebellar cortex, and not for small granule neurons (Figure 3). Obesity or high fat diet results in the accumulation of senescent cells in the mouse brain ([1, 20]. We found a SA-β-gal staining pattern in the cerebellum of 9 weeks old adult leptin receptor deficient *ob/ob* mice that was identical to the one observed in wild type mice (Supplementary Figure 1), thus obesity-independent. Next, we tested the positivity of Purkinje cell layer for β-gal and for other established positive (p16, p21 and DPP4 [19, 21–24]) and negative (H3K4me3 [25]) markers of cell senescence by immunofluorescence staining in wild type mice, as recommended recently to verify a senescent phenotype [1]. Surprisingly, in the cerebellum, Purkinje cells were strongly immunopositive for p16, p21 and DPP4 expression, while they were negative for H3K4me3 that instead stained intensely small granule neurons (Figure 7). Within the central nervous system (CNS) of wild type mice, we detected a distinct β-gal staining at pH 4 and SA-β-gal staining at pH 6 specifically in the choroid plexus, which produces the cerebrospinal fluid in the ventricles of the brain and consists of modified ependymal cells (Figure 4). The observed β-galactosidase staining pattern was identical in the choroid plexus of *ob/ob* mice (Supplementary Figure 2). Ependymal cells were found strongly immunopositive for p16, p21 and DPP4 expression, while staining for H3K4me3 was present but irregular and patchy compared to the other markers (Figure 8). Altogether, our findings demonstrate *a bona fide* senescent-like phenotype of adult mouse cell types/tissues (Purkjnie cell layer, choroid plexus, pancreatic islets and intestinal crypts), based on SA-β-gal staining and other markers of senescence.

## DISCUSSION

Endogenous β-galactosidase staining (pH 4) has been described in murine pancreatic islets [10]. To our knowledge ours is the first study reporting SA-β-gal in pancreatic islets: cells residing in the islets of Langerhans are terminally differentiated cells and almost entirely in a post-mitotic state [26]. Positive SA-β-gal staining in intestinal crypts is in agreement with previous findings showing increasing γH2AX foci-positive crypt enterocytes in old mice [27–29]; in this respect, SA-β-gal positive cells within the intestinal crypts could be Paneth cells, which are entirely post-mitotic cells [30]. Future studies of colocalization of SA-β-gal staining and Paneth cells specific markers (i.e lysozyme) will shed light on this issue.

Post-mitotic cells are essential for the function of the brain. Recently, it was shown that Purkinje cells from old (32 months of age), but not from young (4 months of age), displayed oxidative stress, γH2AX and SA-β-gal staining: a co-localisation of multiple senescence markers in the same neurons [31]. This was the first study describing a “senescence-like phenotype” in post-mitotic cells of old, but not young, healthy mice. Our data are fully consistent and demonstrate that the senescence-like phenotype of Purkinje cell layer might actually start before middle age in mice, as middle age in mice is considered to start around 10 months of age [32]. Low bacterial β-gal staining was previously detected in the choroid plexus in mice [33, 34]. Our data on SA-β-gal stained choroid plexus are reminiscent of those showing increased expression of markers of senescence, particularly those related to obesity-induced inflammation, in the periventricular area adjacent to the lateral ventricle, which is located near the root of the choroid plexus [20]. Choroid plexus produces CSF and participate in brain immunosurveillance. During ageing, CSF secretion decreases as much as 50%. These modifications are concurrent with subnormal brain activity, reduced beta-amyloid clearance, and increased glycation phenomena as well as oxidative stress [35]. The potential interplay between senescent phenotype of the choroid plexus at young/mid age and its functional decline at older age is unknown. Senescence markers have been observed in neurons in the CNS also in a pathological context, during ischemia or Alzheimer’s disease [36, 37].

Accumulation of multiple senescence markers in aging mice has been shown for major post-mitotic cells types residing in different tissues, such as retinal ganglion cells, cardiomyocytes, skeletal myofibres, cochlear cells and osteocytes (reviewed in [3]) The physiological or aging role of the potential senescent phenotype – as we identified by SA-β-gal, p16, p21 and DPP4 marker expression levels - in the different functions performed by Purkinje cells and ependymal cells, specialized post-mitotic neuronal cell types positive, in not aged healthy mice remains unclear. During rat post-natal cerebella development, the period of maximal differentiation between days 9 to 13 was associated with a change in p21 and p16 staining from the external granular and Purkinje cells to a primarily Purkinje cell distribution [38]. Expression levels of H3K4me3 and DPP4 and their signaling pathways have been implicated in several aspects of murine brain homeostasis, from development to aging [39–42]. To our knowledge, ours is the first report to detect expression of DPP4 in Purkinje cells; DPP4 inhibitors are new promising therapeutic approach against Alzheimer’s disease [41]. Future research on senescent post-mitotic cells should encompass also the crucial role of mammalian target of rapamycin (mTOR) pathway. During cell cycle arrest caused by contact inhibition cells do not undergo a fully senescent phenotype. It was demonstrated that the conversion from cell cycle arrest to senescence, a phenomenon called geroconversion, requires stimulation of mTOR and downstream effectors, such as pS6K, concomitantly to p16/p21 activation [43, 44]. Therefore, our study thus encourages exploring the function of post-mitotic cells positive for SA-β-gal activity and other senescence markers in healthy adult or middle age organisms, by simultaneous assessment of related phenomena (including SASP, the last step of the proposed multi-marker, three-step workflow for detecting senescent cells [1]), to understand whether post-mitotic senescence plays a significant role as driver of ageing phenotypes.

## MATERIALS AND METHODS

### Mice

The C57BL/6 mice strains were purchased from AnLab, Czech Republic. All animal work was conducted in accordance with Act No 246/1992 Coll., on the protection of animals against cruelty under the supervision of the Central Commission for Animal Welfare, approval 39197/2018-MZE-17214. Mice were housed under controlled conditions (light-dark cycle 12 h, 21 ± 2 °C, 40–50% humidity) with food and water available ad libitum. Obese (*ob/ob*) male mice in BL6 background (Charles River, MA, US) were housed in the University of Brescia animal facility (Brescia, Italy). Mice of 9-weeks-of-age were then euthanized and organs were removed. The experimental protocol n°516/2018-PR granted was approved by the University of Brescia Institutional Animal Care Committee (Brescia, Italy) and was conducted in accordance with national and European regulations. After mice sacrifice all tissues were collected, washed in PBS, fixed in 4% paraformaldehyde for 24h and de-hydrate in sucrose 5% at least for 24hr. Finally, they were embedded in OCT and sectioned in 10μM slices with a frozen microtome.

### Histochemistry

B-galactosidase detection method was performed as previously described [18, 19]. Briefly, tissues sections were fixed in 1% formalin in PBS for 1 min at RT, washed three times in PBS and incubated overnight on X-gal staining solution [1 mg/mL of X-gal (VWR), 40 mM citric acid/sodium phosphate buffer, 5 mM potassium ferricyanide (Sigma), 5 mM potassium ferrocyanide (Sigma), 150 mM NaCl, and 2 mM MgCl2] at 37°C in a humidified chamber. The experiments were carried out using staining solutions at different pH (from 4 to 7) to assess the SA-β-gal activity. Samples were rinsed with distilled water and counterstained with Nuclear Fast Red (Sigma) for 5 minutes. Images were acquired using Pia-Apochromat 20x 0.8 M27objective on Axio scan Z1 (Zeiss).

### Immunofluorescence

Immunofluorescence staining was performed on mice tissues sections as previously described [45–48]. Mice tissues sections were re-hydrate in PBS for 10 min and treated with the TrueBlack Autofluorescence quencher (Biotium) for 30 sec. After careful washing in PBS, sections were blocked for 60 min in M.O.M blocking solution (Vector Laboratories) and then incubated with primary antibody overnight. Two primary antibodies co-staining solutions were used on adjacent sections to detect target proteins: one mix containing p21 (ab80633), B-gal (ab9361) and H3K4me3 (ab213224); and another mix containing CDKN2A/p16INK4a (ab189034) and anti-DPPIV/CD26 (R&D Systems MAB1180). All the antibodies were used at 1:500 dilution except for anti-DPPIV/CD26 that was used at 1:1000. The staining was developed using Alexa fluorescent (488, 555, 647) conjugated secondary antibodies, and images were acquired using Axio scan Z1 (Zeiss).

### Data analysis

Image analysis was performed using ImageJ (http://rsb.info.nih.gov/ij/), ZEN 2011 SP1 (black edition) version 8.1., or ZEN 2 version 2.0.0.0. (Carl Zeiss Microscopy GmbH).

Positivity for each marker was decided if the cellular signal intensity was clearly above background intensity seen in the isotype negative controls. Greater than 80 Purkinje cells and > 200 choroid plexus cells were counted per animal and stained marker. Data were presented as means ± SD of 3 animals for group.

## Supplementary Material

Supplementary Figures
