# Supplementary material for: Senescence-like phenotype in post-mitotic cells of mice entering middle age
Source: Aging (Albany NY). 2020 Jul 31;12(14):13979–90. doi: 10.18632/aging.103637 (PMC7425512; doi:10.18632/aging.103637)
Supplement: Supplementary Figures [file aging-12-103637-s001..pdf]

SUPPLEMENTARY FIGURES

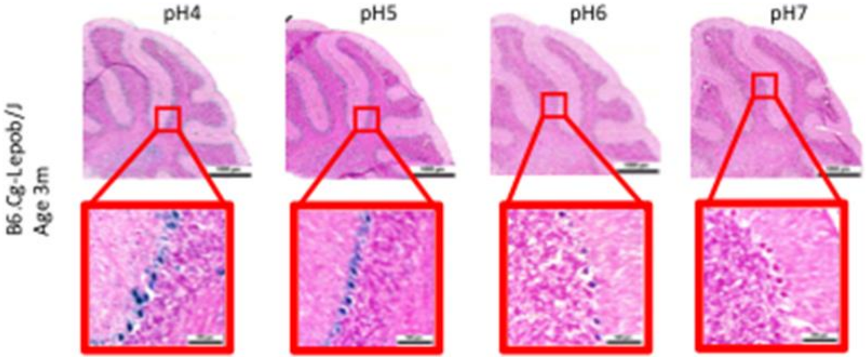

Supplementary Figure 1. pH-dependent (pH 4 to pH 7) β-gal activity in frozen sections of mouse cerebellum from 3 month old leptin receptor-deficient *ob/ob* mice. Nuclear Fast Red was used for counterstaining. At pH 6, specific for SA-β-gal, bluish color from β-gal activity is evident specifically in the Purkinje cell layer. Representative images from 3 different mice per genotype are shown.

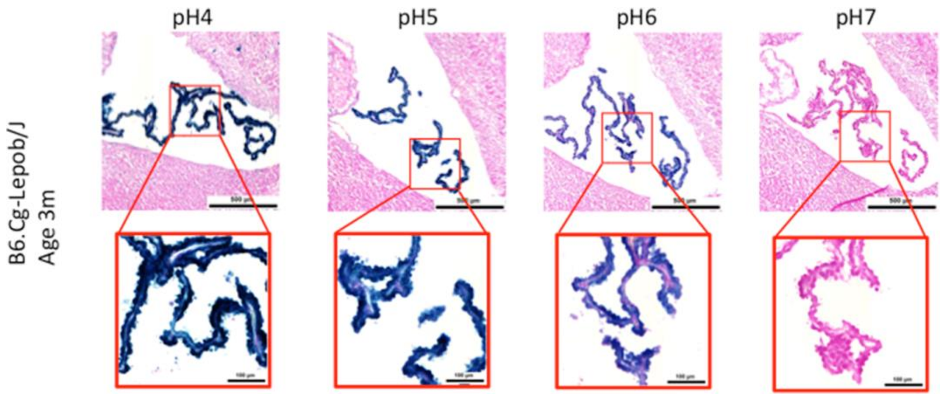

Supplementary Figure 2. pH-dependent (pH 4 to pH 7) β-gal activity in frozen sections of choroid plexus from 3 months old leptin receptor-deficient *ob/ob* mice. Nuclear Fast Red was used for counterstaining. At pH 6, specific for SA-β-gal, bluish color from β-gal activity is evident specifically in ependymal cells in the choroid plexus. Representative images from 3 different mice are shown.
